# Gender disparities in a psychiatric department in Tunisia

**DOI:** 10.1192/j.eurpsy.2022.2240

**Published:** 2022-09-01

**Authors:** E. Bergaoui, M. Zrelli, N. Staali, M. Moalla, R. Lansari, A. Larnaout, W. Melki

**Affiliations:** Razi Hospital, Psychiatry D, Manouba, Tunisia

**Keywords:** gender disparities, Tunisia

## Abstract

**Introduction:**

Gender disparities exist regarding prevalence, symptomatology and risk factors of mental disorders. In Tunisia, there is only one hospital dedicated entirely to mental health which is Razi hospital.

**Objectives:**

The aim of the present study was to assess gender based mental health disparities in a psychiatric department and its related factors.

**Methods:**

A cross sectional and comparative survey was conducted between March and April 2021 in the department of psychiatry D of Razi Hospital including 70 patients with a sex ratio= 1.

**Results:**

The participants were aged between 17 and 68. Men had higher rate of celibacy: 80% of men against 48.57% of women (p=0.009). A total of 11.42% of women were illiterate against 2.85% of men, 48% of men were unemployed against 62.85% of women. There was a significant difference between gender and use of cigarettes, cannabis and alcohol (p<0.001). The diagnosis was mood disorders for 35.42% of women and 17.14% of men and schizophrenia for 57.14% of women and 77.14% of men. Gender and modality of hospitalization were significantly associated (p=0.046): 14% of women were involuntary hospitalized against 40% of men. Time between symptoms onset and consulting is 3.5 years (±5.67) for women and 1.77 (±4.75) for men. The mean number of admissions for women is 1.59 and for men 4.2 (p=0.009).

**Conclusions:**

Onset of mental disorders for women is 3 to 4 years later than men. They have better premorbid functionning and better social networks.Gender disparities are not only determined biologically but also socially.

**Disclosure:**

No significant relationships.

